# Safety, tumor trafficking and immunogenicity of chimeric antigen receptor (CAR)-T cells specific for TAG-72 in colorectal cancer

**DOI:** 10.1186/s40425-017-0222-9

**Published:** 2017-03-21

**Authors:** Kristen M. Hege, Emily K. Bergsland, George A. Fisher, John J. Nemunaitis, Robert S. Warren, James G. McArthur, Andy A. Lin, Jeffrey Schlom, Carl H. June, Stephen A. Sherwin

**Affiliations:** 1 Cell Genesys, Inc, Foster City, CA USA; 2 Celgene Corporation, San Francisco, CA USA; 3 University of California, San Francisco, CA USA; 4 Stanford University, Stanford, CA USA; 5 Mary Crowley Cancer Center, Dallas, TX USA; 6 Laboratory of Tumor Immunology and Biology, Center for Cancer Research, National Cancer Institute, Bethesda, MD USA; 7 University of Pennsylvania, Philadelphia, PA USA

## Abstract

**Background:**

T cells engineered to express chimeric antigen receptors (CARs) have established efficacy in the treatment of B-cell malignancies, but their relevance in solid tumors remains undefined. Here we report results of the first human trials of CAR-T cells in the treatment of solid tumors performed in the 1990s.

**Methods:**

Patients with metastatic colorectal cancer (CRC) were treated in two phase 1 trials with first-generation retroviral transduced CAR-T cells targeting tumor-associated glycoprotein (TAG)-72 and including a CD3-zeta intracellular signaling domain (CART72 cells). In trial C-9701 and C-9702, CART72 cells were administered in escalating doses up to 10^10^ total cells; in trial C-9701 CART72 cells were administered by intravenous infusion. In trial C-9702, CART72 cells were administered via direct hepatic artery infusion in patients with colorectal liver metastases. In both trials, a brief course of interferon-alpha (IFN-α) was given with each CART72 infusion to upregulate expression of TAG-72.

**Results:**

Fourteen patients were enrolled in C-9701 and nine in C-9702. CART72 manufacturing success rate was 100% with an average transduction efficiency of 38%. Ten patients were treated in CC-9701 and 6 in CC-9702. Symptoms consistent with low-grade, cytokine release syndrome were observed in both trials without clear evidence of on target/off tumor toxicity. Detectable, but mostly short-term (≤14 weeks), persistence of CART72 cells was observed in blood; one patient had CART72 cells detectable at 48 weeks. Trafficking to tumor tissues was confirmed in a tumor biopsy from one of three patients. A subset of patients had ^111^Indium-labeled CART72 cells injected, and trafficking could be detected to liver, but T cells appeared largely excluded from large metastatic deposits. Tumor biomarkers carcinoembryonic antigen (CEA) and TAG-72 were measured in serum; there was a precipitous decline of TAG-72, but not CEA, in some patients due to induction of an interfering antibody to the TAG-72 binding domain of humanized CC49, reflecting an anti-CAR immune response. No radiologic tumor responses were observed.

**Conclusion:**

These findings demonstrate the relative safety of CART72 cells. The limited persistence supports the incorporation of co-stimulatory domains in the CAR design and the use of fully human CAR constructs to mitigate immunogenicity.

## One sentence summary

The authors describe the first human application of autologous chimeric antigen receptor gene-modified T cells targeting TAG-72 in the treatment of metastatic colorectal cancer in two clinical trials.

## Background

Adoptive transfer of T lymphocytes to target and treat cancer is a field that has been evolving over the past 25 years. Initial efforts focused on isolation and expansion of bulk T cells from peripheral blood or tumor infiltrates, expansion ex vivo in the presence of stimulatory cytokines and re-infusion into cancer patients [1]. These efforts suffered from a lack of defined tumor specificity and inability to track the fate of transferred cells. In the 1990s several new approaches were explored in parallel, including genetic modification of T cells to express known tumor-specific αβ T-cell receptors (αβ TCR) and genetic engineering of T cells to express chimeric antigen receptors (CARs) [2]. The latter approach employed engineered CARs composed of an external antigen-binding domain, typically a single-chain variable fragment (scFv) targeting a tumor cell surface antigen, linked to a transmembrane domain and an intracellular-signaling domain, initially limited to the zeta (ζ) chain of the TCR complex [3], and more recently including additional signaling domains such as CD28 and 4-1BB [4, 5]. Stable introduction of CARs into T cells has focused on integrating viral vectors, including gamma retroviruses [6] and lentiviruses [7], to enable in vivo expansion and persistence of gene-modified T cells. CAR-T cell approaches offer the advantage of being human leukocyte antigen unrestricted, but are limited to targeting molecules expressed on the extracellular tumor cell surface.

Clinical investigation of CAR-T cells was undertaken by Cell Genesys, Inc in the mid-1990s. The initial programs to enter the clinic focused on CD4-ζ-modified CAR-T cells for treatment of human immunodeficiency virus (HIV) infection [8]. A series of trials were conducted starting with the treatment of HIV-infected discordant, syngeneic twin pairs in which CD8 T cells were isolated from the uninfected twin, genetically modified with the CD4-ζ CAR and re-infused into the HIV-infected twin [9]. This was followed by a series of trials of autologous CD4-ζ-modified CAR-T cells exploring different CAR-T cell populations (mixed CD4 and CD8 T cells), optimized CAR-T cell ex vivo activation methodologies utilizing immunomagnetic beads coated with antibodies to CD3 and CD28 (CD3xCD28 beads) [10], in vivo CAR-T cell treatment with and without interleukin-2 (IL-2), and treatment of both high-viral-burden HIV disease and minimal residual disease [11, 12]. Results from these trials suggested that both ex vivo stimulation of CAR-T cells with CD3xCD28 beads, and co-infusion of CAR-modified CD4 and CD8 T cells were important for long-term CAR-T cell persistence. No immunogenicity directed against the native human CD4 protein extracellular domain of the CD4-ζ CAR-T cells was observed and stable persistence of infused CD4-ζ CAR-T cells was confirmed for up to 10 years [13].

Concurrent with these trials in HIV infection, CAR-T cell approaches targeting solid tumors were also pursued. T cells expressing scFv-based CARs had demonstrated preclinical activity against a variety of tumor targets [14–16]. The initial solid tumor-associated CAR target selected for clinical application was TAG-72, an oncofetal mucin overexpressed by most human epithelial adenocarcinomas, with expression predominantly restricted to tumor cells [17]. Prioritization of this target was also influenced by the availability of a humanized antibody directed against TAG-72 (huCC49) [18]. This huCC49 antibody was created by grafting the complementarity-determining regions (CDR) from the mouse CC49 (muCC49) antibody [19] onto a human framework. In addition, there existed a substantial body of human safety data following administration of the radiolabeled muCC49 in over 500 patients as a radiotherapeutic [19–21] or radiodiagnostic tool [22]. Human anti-mouse antibody (HAMA) responses associated with rapid clearance of repeat doses of muCC49 were commonly observed in these clinical trials. Use of a humanized scFv CC49 CAR construct was deemed preferable to reduce the risk of antibody induction against a murine scFv-based CAR and potentially avoid immunogenic clearance of TAG-72 targeted CAR-T cells.

A gamma-retroviral CAR construct was created consisting of an extracellular domain composed of an scFv derived from the huCC49 antibody linked to an IgG1 hinge, CH3 spacer, human CD4 transmembrane domain and human CD3-ζ intracellular-signaling domain. This vector was used to transduce CD4 and CD8 T cells using the *kat*293 system [6] to create CC49-ζ-modified T cells targeting TAG-72 [23]. CAR-T cells cultured in the presence of anti-CD3 (OKT3) plus IL-2 were shown to become unresponsive to repeated stimulation through the CC49-ζ CAR, whereas CAR-T cells cultured in the presence of CD3 and CD28 co-stimulation remained responsive to repeated rounds of IL-2-induced proliferation. CC49-ζ CAR-T cells secreted immune-reactive cytokines (granulocyte-macrophage colony-stimulating factor, interferon [IFN]-γ, tumor necrosis factor-α), proliferated, and killed tumor cell lines as well as primary human colorectal tumors expressing TAG-72 in vitro. CC49-ζ-modified CD4 and CD8 T cells were capable of killing TAG-72-expressing tumor cells with equivalent efficiency, but only CD4 CAR-T cells were capable of IL-2 secretion and proliferation following CD3 and CD28 co-stimulation, suggesting that both populations were necessary for optimal in vivo proliferation and tumor killing [23]. Since TAG-72 is secreted at different levels in the sera of patients with advanced TAG-72-expressing tumors [24], additional studies were performed that confirmed a lack of inhibition of CC49-ζ CAR-T cell activity in the presence of soluble TAG-72 protein. The activity of CC49-ζ CAR-T cells was confirmed in vivo in a subcutaneous and disseminated TAG-72-expressing tumor model [23].

Based on these encouraging preclinical data, two clinical trials investigating CC49-ζ-expressing CD4 and CD8 CAR-T cells stimulated ex vivo with CD3xCD28 immunomagnetic beads (hereafter referred to as CART72 cells) in patients with metastatic colorectal cancer (CRC) were initiated in 1997. In the first trial (C-9701), patients received escalating doses of CART72 cells administered by intravenous (IV) infusion. Since the majority of the blood supply to hepatic tumors is derived from the hepatic artery (HA), and to avoid potential trapping of CART72 cells in the lung following IV administration, a second trial (C-9702) was initiated in CRC patients with liver metastases in which CART72 cells were infused directly into the HA. Given the known heterogeneous expression of TAG-72 in metastatic CRC and the ability of interferons to upregulate expression of this antigen [22], it was elected to co-administer IFN-α with each dose of CART72 cells in both trials.

Given the current resurgence in interest in cancer directed CAR-T cells, the now historical results of these first two seminal clinical trials investigating first-generation CAR-T cells in solid tumors are reported here. These data provide important insights into some of the challenges associated with the use of CAR-T cells to treat solid tumors including tumor trafficking, CAR-T cell persistence and anti-CAR immunogenicity.

## Methods

### Clinical trial design and planned treatments

Two open-label, phase 1 trials of CART72 cells in patients with metastatic CRC with liver metastases were conducted between 1997 and 1998 under sponsorship by Cell Genesys, Inc. CART72 cells were administered by IV infusion in trial C-9701 and by intra-HA infusion in trial C-9702. Eligible patients in both trials were adults with metastatic CRC, including at least one liver metastasis, with Karnofsky performance status of ≥ 80%, total bilirubin < 3 times the upper limit of normal (ULN), and transaminases < 5 times ULN. Additional eligibility included expression of TAG-72 in ≥ 5% of tumor cells as detected by immunohistochemistry with the CC49 antibody and serum TAG-72 levels < 500 U/mL. Patients who received prior treatment with immunotherapy, retroviral gene therapy or CC49 antibody were excluded. The primary endpoint was safety and secondary endpoints included tumor response, TAG-72 upregulation with IFN-α, CART72 persistence in blood and tumor, and anti-CAR immunogenicity.

In the first trial (C-9701), six patients were scheduled to receive intra-patient dose escalations of up to five infusions of CART72 cells administered by IV infusion at 2-week intervals at two U.S. sites (UCSF and Stanford). Planned doses included 10^8^, 10^9^, 10^10^, 10^10^ and 10^10^ total T cells with doses calculated based on total CD3 T cells rather than transduced CART72 cells (Part A). This was followed by four patients with three planned biweekly IV infusions of 10^10^ total T cells (Part B). In trial C-9702, six patients with CRC with liver metastases were planned to receive intra-patient dose escalations of 10^9^, 10^10^, 10^10^ and 10^10^ total T cells (doses also calculated based on total CD3 T cells rather than transduced CART72 cells) administered via direct intra-HA infusion at two U.S. sites (UCSF and Mary Crowley Cancer Center). All patients in trial C-9702 were required to have pre-existing percutaneous HA catheters and infusion pumps in place from previous HA infusion chemotherapy protocols. All patients in both trials received co-administration of IFN-α at a dose of 3 million units (MU) by subcutaneous injection every other day for four doses with each CART72 infusion in an effort to upregulate expression of tumor-associated TAG-72. No lymphodepleting chemotherapy was given prior to CART72 administration.

### Response and toxicity evaluation

Tumor response was measured using standard World Health Organization (WHO) criteria; immune-related response criteria had not yet been defined [25]. Safety evaluation included standard monitoring using Common Terminology Criteria for Adverse Events (CTCAE v2.0). Adverse events were assessed as not related, possibly or probably related to CART72 cells and/or IFN-α.

### *Preparation and testing of CC49-ζ Vector and CART72 Cells*

**Fig. 1:**
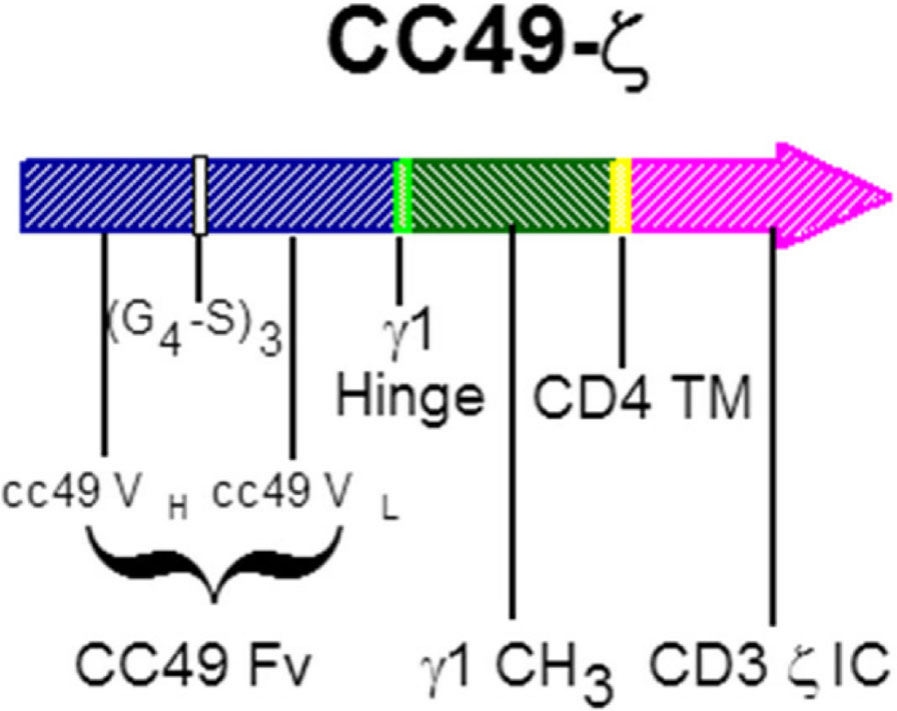
CC49-ζ vector diagram. A gamma retroviral CAR construct consisting of a TAG-72 antigen-binding domain derived from the humanized CC49 single-chain antibody linked to the CD3-ζ signaling domain of the T-cell receptor via a human CD4 transmembrane domain. CAR, chimeric antigen receptor; Fv, variable fragment; (G_4_-S)_3_, (Gly_4_, Ser)_3_ peptide linker; IC, intracellular; TM, transmembrane; V_H_, variable heavy chain; V_L_, variable light chain; γ1, immunoglobulin γ1

Generation of the CC49-ζ retroviral vector has been described previously [23]. The vector diagram is shown in Fig. 1 and includes the huCC49 scFv linked to an IgG1 hinge and CH3 spacer, CD4 transmembrane domain and CD3zeta signaling domain. The clinical grade, high titer (>10^6^ infectious units/mL), CC49-ζ retrovirus vector used for ex vivo transduction of patient lymphocytes was produced under Good Manufacturing Procedures at Cell Genesys using the *kat*293 production cell line [6]. Master cell bank and vector lots all tested negative for RCR by supernatant amplification and co-cultivation on *Mus dunni* cells [26]. Patient lymphapheresis was performed at the participating clinical sites using an automated cell separator (Cobe Spectra or CS-3000, Lakewood, CO, USA) to achieve a minimum of 5 x 10^9^ PBMCs in approximately 300 mL final volume. Apheresis products were shipped to Cell Genesys, Foster City, CA, USA, and processed within 24 hours of receipt in a closed system using Ficoll density gradient separation with a Stericell device (Haemonetics, Braintree, MA, USA) to remove residual red blood cells. Recovered PBMCs were stimulated using immunomagnetic beads (Dynal, Oslo, Norway) coated with anti-CD3 (OKT3) and anti-CD28 (monoclonal antibody 9.3), at a bead to cell ratio of 3:1, in serum-free AIM-V medium (Gibco, Long Island, NY, USA) and IL-2 (200 IU/mL; Chiron, Emeryville, CA, USA). On Day 3, the beads were removed using a Maxsep magnetic bead separator (Baxter, Roundlake, IL, USA) and resuspended in AIM-V medium with IL-2 (200 IU/mL). Transduction with CC49-ζ retroviral supernatant was performed on Days 5 and 7 in AIM-V medium containing IL-2 and polybrene (Aldrich, St Louis, MO, USA) using spinoculation at 800 x g for 30 minutes. After transduction, cells were expanded in AIM-V medium with 200 IU/mL IL-2 in LIFECELL 3-L bags (Baxter) until the target cell dose of > 4 x 10^10^ T cells was obtained (Days 10–17). The final CART72 cell products were cryopreserved in 50 mL sterile bags containing 6 x 10^9^ cells/bag in Plasmalyte-A (Baxter IV Systems, Roundlake, IL, USA) with 10% dimethylsulfoxide (Sigma, St Louis, MO, USA), 1% dextran-40 (Baxter IV Systems), and 5% human serum albumin (Alpha Therapeutics, Los Angeles, CA, USA), and stored in liquid nitrogen. Final T-cell products were tested for viability (by trypan blue exclusion), sterility, mycoplasma, transduction efficiency by flow cytometry using anti-idiotypic antibody (AI49-3; as described previously) [23], RCR (co-cultivation on *M. dunni* cells) [26], and TAG-72-specific ^51^Cr release cytolytic activity against 293 cells (human embryonic kidney line) expressing TAG-72. Phenotypic characterization was performed using antibodies to CD3, CD4, CD8, CD28 (Coulter, Miami, FL, USA), CD25 (Caltag, Burlingame, CA, USA), and CD62L (Pharmingen, San Diego, CA, USA) by flow cytometry. Mouse IgG control was used to determine background staining and to set percent positive values for each of the CD markers. Cryopreserved CART72 cells were thawed in a 37 °C water bath at the patient’s bedside and infused directly through an IV or intra-HA catheter over 5–10 minutes per bag. Infused CART72 cell dose was calculated based on total T cells rather than transduced cells.

### TAG-72 radioimmunoassay and immunogenicity assessment

Monitoring of serum TAG-72 levels in treated patients was originally planned using a CC49 capture RIA (Centocor 72–4 RIA). Following preliminary data suggestive of artefactual drops in TAG-72, this assay was modified to identify the presence of anti-idiotype antibody induction against the TAG-72 binding epitope of CC49. Enzyme immunoassay plates were coated with two non-competing murine TAG-72 binding antibodies (muCC49 and B72.3) at 10 μg/mL. Plates were blocked with Blocking Buffer (2% dry milk, PBS 0.05%, Tween20) and washed with Wash Buffer (PBS 0.05%, Tween 20). Normal Human Serum (NHS), patient serum with confirmed anti-idiotype reactivity to CC49, and the Blocking Buffer were run as controls. An anti-idiotype blocking antibody was not available for B72.3. Patient serum and controls were diluted in the Blocking Buffer at 1:100, 1:1000 and 1:10,000, and were added to the plate. Plates were washed and the detector antibody, polyclonal goat anti-human Ig labeled with HRP diluted 1:5000 in PBS was added. The plates were washed again and developed using o-phenylene-diamine-dihydrochloride and optical densities were measured at 490 nm. Positive anti-idiotypic CC49 antibody activity was considered when measurements were at least 1.8 times higher than NHS at the same dilution.

### Patient serum mixing studies and heat extraction

Patient serum was assessed for the presence of a TAG-72 binding inhibitor in the Centocor 72.4 RIA. Sera were analyzed prior to and following abrupt drops in measured TAG-72 levels (after repeated infusions of 10^10^ CART72 cells). Sera from the early time points were mixed in equal volumes with NHS or with sera from the late time points. Mixtures were mixed gently for 1 hour at room temperature and then assayed in the TAG-72 capture RIA. Sample mixtures that showed a significant reduction in titer when compared to NHS mixtures were considered positive for a CC49 or TAG-72 inhibitor. Heat treatment of serum was used to precipitate Ig in conditions designed to not affect TAG-72 antigen immunoactivity. Patient sera samples were diluted 1:3 in 0.01 M Bis Tris 0.15 M sodium chloride (pH 6.5). Samples were split into two sets. One set was run as is (diluted 1:3 with no heat treatment) and the other set was boiled for 5 minutes at 90–100 °C. Both sample sets were centrifuged at ~2000 g for 5 minutes. Supernatants were tested in the TAG-72 capture RIA for quantification of TAG-72.

### CC49-ζ quantitative PCR assay

CART72 DNA levels in circulating PBMC and tumor biopsy samples were measured using methods previously described [11] using primers specific for the CC49-ζ construct. Planned time points included screening, Day 0 and 1 of each CART72 infusion, then every 2 weeks for the first 12 weeks and quarterly thereafter for one year.

### RCR Testing by PCR

Analysis for RCR was performed using Taqman PCR as previously described [11]. RCR testing was performed on released CART72 products and testing was planned on blood samples from all enrolled patients at screening, Week 4, every 3 months for the first year and annually thereafter.

### ^111^Indium-labeled T-cell substudy 

An Indium labeling substudy was performed in both trials to explore trafficking of CART72 cells following IV and intra-HA administration [27, 28]. The ^111^Indium labeling procedure was planned during one of the 10^10^ CART72 cell infusions. Cryopreserved final product CART72 cells were aliquoted in bags containing 1–5 x 10^9^ cells each, and only one bag was used for the ^111^Indium-labeling procedure. On the day of the scheduled CART72 cell infusion, one bag of CART72 cells was thawed at the clinical site, transferred on ice to the Nuclear Medicine department at each institution, and labeled with 500–700 mCi of sterile ^111^Indium-oxyquinolone at room temperature for 10–20 minutes with gentle rocking. A 5–10 mL aliquot of the labeled cell suspension was removed for cell counting, determination of cell viability by trypan blue exclusion and labeling efficiency. All manipulations during the cell labeling were carried out under aseptic conditions using standard techniques employed in routine ^111^Indium-labeling procedures. Unlabeled CART72 cells were infused prior to the infusion of ^111^Indium-labeled cells. Infusion occurred within 1–3 hours of the labeling procedure and receipt of the unlabeled CART72 cells. Following infusion, the infusate bag and tubing were returned to the Nuclear Medicine department for appropriate disposal as radioactive waste. ^111^Indium has a radioactive half-life of 67 hours. The estimated total body radiation exposure to the patient was 0.31 rads/500 mCi of ^111^In. In addition, standard Technicium aggragated albumin (^99m^Tc-MAA) was infused concurrent with ^111^Indium-labeled CART72 cells to visualize vascularized liver and tumor tissue. Analog and digital gamma camera images were obtained at 3–6 hours after infusion and again at 24, 48, 72 and 96 hours post-infusion. At the discretion of the Principal Investigator additional images were allowed on additional days up to 15 days post-infusion.

### Tumor biopsies

Optional percutaneous, ultrasound or computed tomography-guided paired tumor biopsies were performed in eligible patients at screening and various time points following CART72 infusion. Percutaneous liver biopsies were preferred, but biopsies of alternative accessible tumor sites were allowed. Planned analyses included routine histopathology, TAG-72 expression by immunohistochemistry using CC49 antibody, and CART72 quantitation by PCR.

## Results

### Patient characteristics and treatment

Between 1997 and 1998, two clinical trials investigating CART72 cells were conducted in patients with metastatic CRC at three clinical sites in the United States. All enrolled patients underwent a lymphapheresis procedure followed by ex vivo culture, retroviral gene modification and expansion of autologous CART72 cells. CART72 cells were administered by IV infusion in trial C-9701 and by direct intra-HA infusion in trial C-9702. In both trials, a brief course of IFN-α was given with each CART72 infusion to upregulate expression of tumor-associated TAG-72.

A total of 14 patients were enrolled in trial C-9701 and nine in trial C-9702, each at two clinical sites. In trial C-9701, all 14 patients underwent lymphapheresis and 10 (71%) initiated treatment with CART72 cells administered by IV infusion (six in Part A; four in Part B). Four patients were not treated because of patient withdrawal (*n* = 2) or death due to progressive disease (*n* = 2) prior to their first infusion. In trial C-9702, nine patients were enrolled and six were treated with intra-HA infusions of CART72 cells. Three patients were withdrawn prior to their first infusion, most likely due to progressive disease, but records are no longer available.

In trial C-9701, five of six patients from Part A completed all five planned IV infusions (10^8^, 10^9^, 10^10^, 10^10^, 10^10^ total T cells) and one withdrew after three infusions (10^8^, 10^9^, 10^10^) because of disease progression. In Part B, two of four patients completed all three scheduled infusions (10^10^ x 3) and two patients received two infusions prior to withdrawal because of disease progression and toxicity, respectively. In trial C-9702, five of six patients received all four planned intra-HA infusions (10^9^, 10^10^, 10^10^, 10^10^ total T cells); one patient received a 50% reduced dose of CART72 cells for the last two infusions and one was withdrawn after the third infusion because of toxicity. Based on the average transduction efficiency of 38%, each 10^10^ total T cell dose contained approximately 3–4 x 10^9^ CART72 cells.

Patients enrolled in trial C-9701 were mostly Caucasian (one African American) of mixed gender (nine male, five female) with a mean age of 56 years and good performance status (Karnofsky score of ≥ 80%). Most had extensive metastatic disease in liver, lung, bone and/or spleen. All patients had been previously treated with surgery and chemotherapy and three had received radiotherapy. All patients enrolled in trial C-9702 had metastatic disease in the liver and had failed prior HA infusional chemotherapy and had HA infusion pumps still in place.

Baseline levels of TAG-72 expression in tumor by immunohistochemistry as well as serum levels of soluble TAG-72 for patients in trial C-9701 are summarized in Table 1. Expression levels were variable and there was poor concordance between tumor expression of TAG-72 and serum levels of TAG-72.

**Table 1:** Baseline serum and tumor TAG-72 levels from trial C-9701

Patient ID	Tumor TAG-72 (% positive)	Tumor TAG-72 Intensity	Serum TAG-72 (U/mL)
201A	5	2+	9.4
202B	60	2+	7.3
203C	95	2+	91.3
204D	5	1+	4.1
205E	80	2+	8.2
206K	60	2+	293.5
207L	70	2+	NA
101F	10	2+	148.3
102G	5	1+	6.4
103H	5	1+	96.9
104I	5	1+	13.6
105J	25	2+	19.8
106M	80	2+	187.4
107N	50	2+	NA

*NA* not available

Tumor TAG-72 levels were measured in archival or fresh tumors at baseline by immunohistochemistry and quantified as percentage of cells staining positive and staining intensity. Serum TAG-72 levels were measured using a CC49 capture radio-immunoassay

### CART72 cell manufacturing

**Fig. 2:**
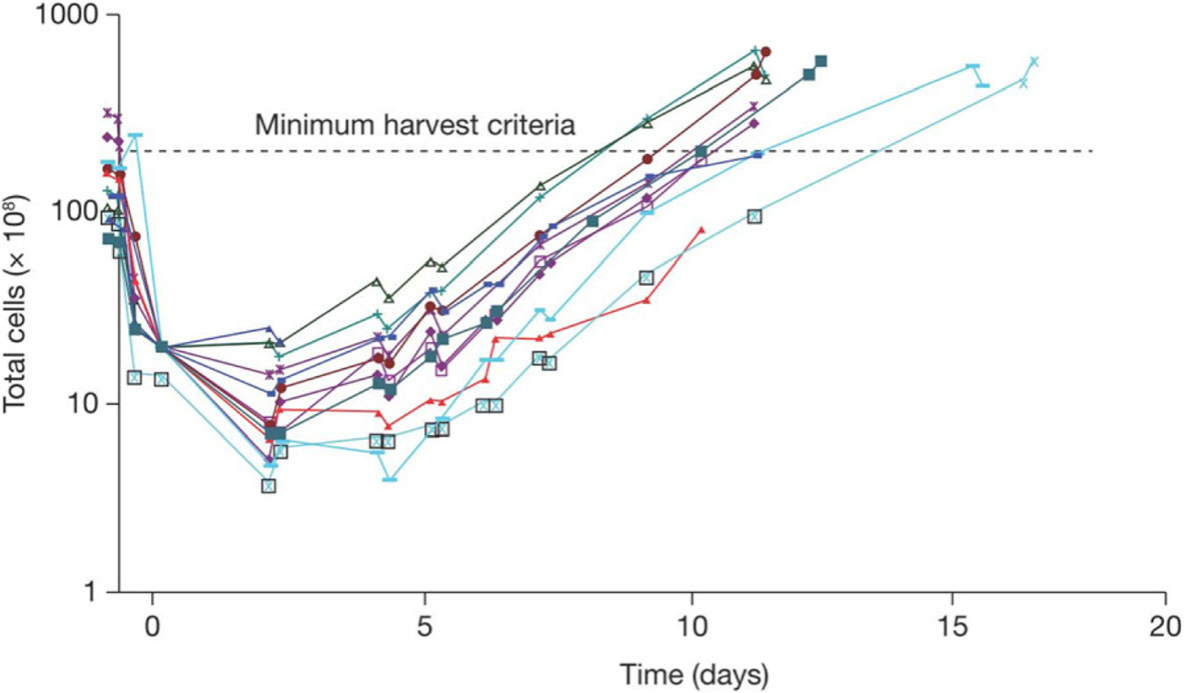
CART72 cell growth curves. Individual CART72 cell growth curves from trials C-9701 and C-9702 are shown. Lymphapheresis products were depleted of red blood cells and stimulated with CD3xCD28 beads on Day 1 in the presence of IL-2 (200 IU/mL) with removal of beads on Day 3. Spinoculation transduction with the CC49-ζ vector was performed on Days 5 and 7 followed by continued cell culture in serum-free media supplemented with IL-2 (200 IU/mL). Minimum harvest criteria of 3 x 10^10^ total T cells was achieved on all lots by Day 17

CART72 cells were successfully processed and released for infusion in all enrolled patients. Unselected autologous T cells from lymphapheresis products were expanded ex vivo after stimulation with anti-CD3 and anti-CD28 antibody conjugated beads (CD3xCD28 beads) in the presence of low-dose IL-2 (200 IU/mL) for an average of 13 days. T cells were transduced with a gamma-retroviral vector containing the CC49-ζ CAR construct (Fig. 1). All CART72 cell cultures were completed within 17 days, reaching the minimum target dose of at least 3 x 10^10^ total CD3 T cells by day 14 (Fig. 2). Average CART72 retroviral transduction efficiency for CD3 T cells as measured by flow cytometry was 38%. CART72 doses for infusion were calculated based on total CD3 T cells rather than transduced cells. The average composition of the final CART72 products was 54% CD4 and 41% CD8 T cells. Percentages of final CART72 cell products expressing CD62L, CD28 and CD25 were 86%, 95% and 50%, respectively. Released CART72 cell products showed potent and specific lysis of cell lines expressing TAG-72 [24] and tested negative for replication competent retroviruses (RCR).

### Safety

Limited safety information is still available from both trials due to incomplete access to historical trial records and complete study reports. In trial C-9701, the most frequently reported grade 1 – 2 adverse events attributed to CART72 cells were chills (70%), fever, dizziness, paresthesia (30% each), headache, tachycardia, myalgia and hypoxia (20% each), all suggestive of low-grade cytokine release syndrome (CRS) or IFN toxicity. Other grade 1 or 2 toxicities attributed to IFN-α included nausea (50%), malaise (40%), flu syndrome, abdominal pain (30% each), tachycardia, anorexia, dry mouth, vasodilation and hypoxia (20% each). No grade 4 related adverse events were reported. Grade 3 adverse events possibly related to CART72 included retinal artery occlusion that developed 3 days following the second CART72 infusion (one patient) and chills (one patient). There were no clinically significant changes in laboratory values that were not directly related to progression of disease. All patients were treated as out-patients and no patients required hospitalization for management of toxicities.

In trial C-9702, one patient (303D) developed fever, abnormal liver function tests, anemia and leukocytosis 6 days following the second CART72 cell administration (first infusion of 10^10^ cells) prompting a 4-day hospitalization. No infection was identified and the patient did not develop hypotension, hypoxia or require vasopressors. Symptoms resolved spontaneously by 12 days post infusion and laboratory abnormalities normalized to baseline levels by 22 days post infusion. The patient went on to receive three additional CART72 cell infusions at a 50% dose reduction of 5 x 10^9^ cells per infusion. These subsequent infusions were well tolerated, but the patient developed a recurrent transient syndrome of fever, increased bilirubin and anemia 2 months following the last infusion, which responded to a short course of prednisone. A second patient (305H) with a history of chronic atrial fibrillation developed fever and rigors 2 hours following the third infusion, complicated by rapid atrial fibrillation, transient hypotension and mild congestive heart failure prompting a 4 day hospitalization. Fevers resolved after 24 hours. This patient also developed transient leukocytosis, anemia and thrombocytopenia with maximal changes 24 – 48 hours post infusion followed by spontaneous recovery. The fourth and final planned CART72 cell infusion was not administered. Infusion-related toxicities that were not dose-limiting were common and included fever, abdominal pain, increased bilirubin, headache, nausea, vomiting and anemia. Five of six patients completed all four planned CART72 cell infusions and no patients died within 30 days of CART72 treatment. RCR tests were negative in all patients through 48 weeks of follow-up in both trials.

### Serum TAG-72 reductions and immunogenicity

**Fig. 3:**
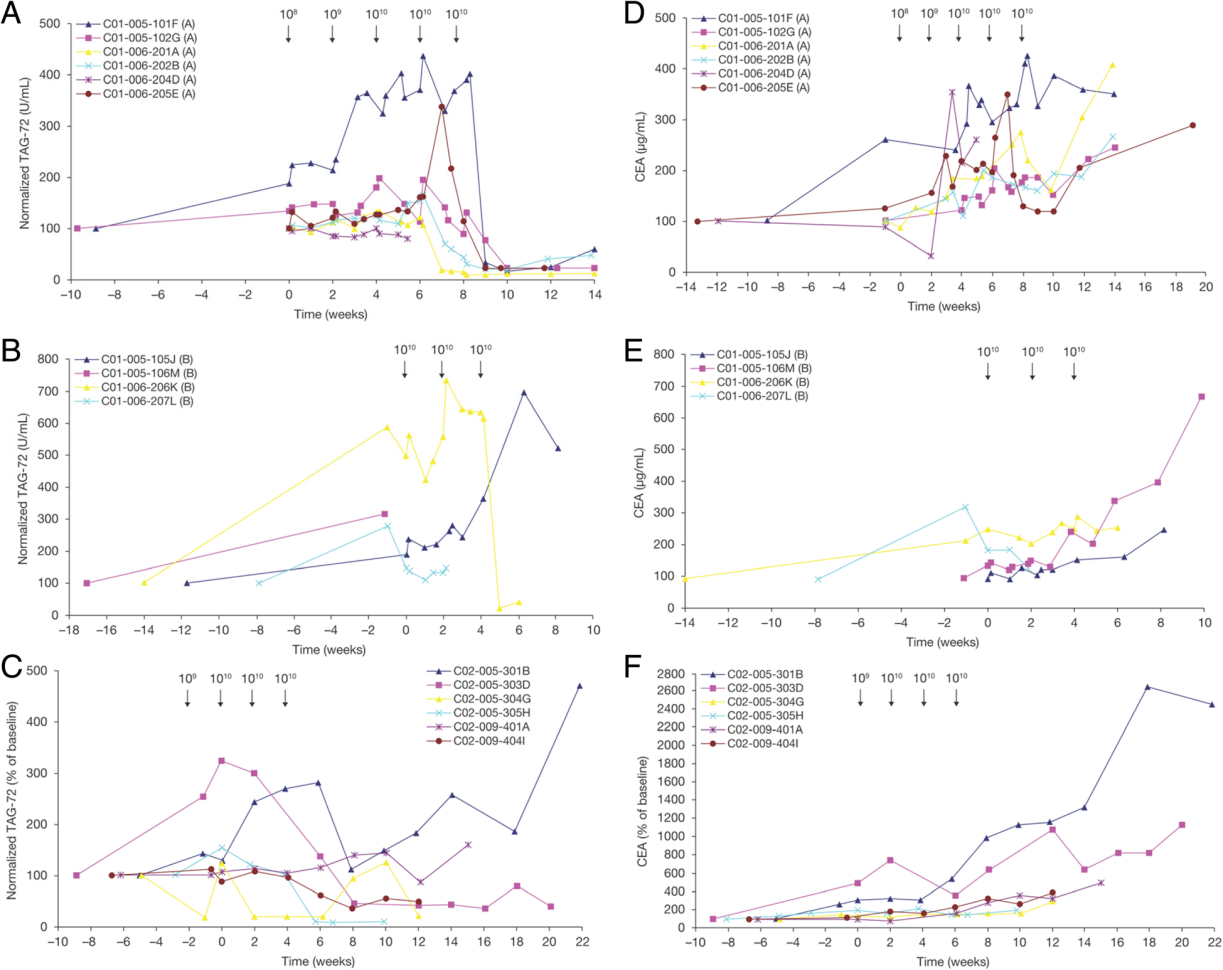
Serum TAG-72 and CEA levels. Circulating sera levels of TAG-72 were measured by RIA in treated patients in: (**a**) Part A dose-escalation phase of IV CART72 cells in trial C-9701; (**b**) Part B expansion phase of trial C-9701; and (**c**) Intra-HA CART72 cells in trial C-9702. Circulating sera levels of CEA were measured in: (**d**) Part A of C-9701; (**e**) Part B of C-9701; and (**f**) C-9702. Seven of 8 evaluable patients treated with IV infusions of CART72 cells later demonstrated abrupt reductions (>80%) in serum TAG-72 levels (**a**, **b**). Two of 2 evaluable patients who received CART72 cells via HA infusion showed a marked decrease in measured serum TAG-72 at Week 8 (85% and 60%, respectively) (**c**). No associated reductions in sera CEA were observed, with an increase in the sera CEA levels over time observed in nine of 10 patients in trial C-9701 (**d**, **e**) and six of six patients in C-9702 (**f**). CEA, carcinoembryonic antigen; HA, hepatic artery; IV, intravenous

Similar to the oncofetal antigen CEA, the TAG-72 mucin is shed into the serum by some proliferating CRC cells [24]. Serum CEA and TAG-72 levels were, therefore, monitored in all patients as potential serum biomarkers of tumor burden and response to CART72 treatment. These test results inadvertently led to the identification of an induced anti-idiotype antibody response directed the CC49-ζ CAR. Serum TAG-72 levels increased acutely in most patients after initiation of IFN-α, suggestive of an IFN-induced increase in TAG-72 expression and/or shedding from tumor or other tissues (Fig. 3). In trial C-9701, seven of 8 evaluable patients with measurable serum TAG-72 later demonstrated abrupt reductions (>80%) in serum TAG-72 levels 5 to 9 weeks following the first cell infusion (Fig. 3a, b). The remaining patient (105 J) showed a 25% decrease at the last time point analyzed (Week 8). Of the two evaluable patients in trial C-9702, both showed a marked decrease in measured serum TAG-72 at Week 8 (85% and 60%) (Fig. 3c).

These large apparent reductions in TAG-72 initially suggested a signal of antitumor efficacy. However all patients demonstrated concurrent radiologic evidence of tumor progression. Furthermore, there were no consistent reductions in CEA at the time of the measured reductions in TAG-72 (Fig. 3d, e, f).

The lack of concordance between TAG-72 levels, CEA levels and tumor radiographic response, suggested that the results from the TAG-72 RIA may have represented an assay artifact. The commercial TAG-72 RIA kit used the muCC49 capture antibody. This raised the possibility of the development of an inhibitory antibody in patient sera that blocked sera TAG-72 binding in the sandwich assay. This hypothesis was confirmed by mixing post-infusion sera from three patients who demonstrated strong post-infusion drops in TAG-72 with TAG-72-positive pre-infusion serum. This resulted in a 59–84% decrease in the measured pre-infusion level of TAG-72 after mixing, consistent with the presence of an inhibitory antibody in the post-infusion sera (data not shown). In contrast, there was no decrease in pre-infusion TAG-72 titer following mixing with normal human sera.

To confirm that the measured post-infusion reductions in TAG-72 were due to assay interference rather than true changes in TAG-72 levels, post-infusion sera samples were heat-treated to denature serum antibodies that might inhibit the detection of TAG-72. Seven of 8 evaluable patients in trial C-9701 and 2 of 2 evaluable patients in trial C-9702, showed an increase in TAG-72 in post-infusion sera following heat treatment, with measured levels similar to the pre-infusion TAG-72 titer (Table 2). These results were consistent with denaturation of the inhibiting serum antibody.

**Table 2:** Anti-CC49-ζ immune responses in patients

Trial	Patient	TAG-72 post heat extraction (fold increase)	Anti-idiotype titer	
			1:100	1:1000
C-9701	101 F	8.1	++	++
	201A	>2.6	+++	+++
	202B	1.8	+++	+++
	206 K	2.5	+	+
	105 J	1.2	+	++
	106 M	2.5	+	+
	204D	UND	-	-
	102G	>1.8	+++	++
	205E	>1.0	++	++
C-9702	301B	1.3	+	++
	303D	>1.4	+	++
	401A	NA	+	-

*NA*, data not available, *UND* TAG-72 undetectable.

+, 1.5- to 3-fold increase; ++, 3.1- to 5-fold increase; +++, > 5-fold increase

TAG-72 was detected in sera samples from IV-infused (trial C-9701) and intra-HA-infused (trial C-9702) CART72 cell patients following heat extraction of the TAG-72 to dissociate the antigen from inhibitory antibodies. Antibodies against the CC49 antibody were detected in all evaluable patients following infusion with CART72 cells comparing the RIA titer post-infusion versus pre-infusion. The detection of anti-CC49 antibodies coincided with the clearance of CART72 cells as measured by PCR

Since muCC49 is the capture antibody in the TAG-72 RIA, possible inhibitors included a human anti-CC49 idiotype antibody that interfered with TAG-72 binding or a human anti-TAG-72 idiotype antibody that interfered with CC49 binding. To further elucidate the nature of the induced inhibitor, pre- and post-infusion sera were next assayed for the presence of anti-CC49 idiotype antibodies using muCC49 as the capture protein. All patients were negative for anti-CC49 reactivity in the pre-infusion sera. In 8 of 10 patients in trial C-9701 and 3 of 3 patients in trial C-9702, post-infusion sera demonstrated an increase in anti-CC49 antibodies, consistent with the induction of an anti-CC49 idiotype antibody (Table 2). To rule out the presence of a TAG-72 immune complex binding to the muCC49 capture antibody, the assay was repeated using B72.3 (another murine antibody recognizing a non-competing epitope of TAG-72) as the capture protein. Seven of 7 patients tested negative in the B72.3 assay in both pre- and post-infusion samples (data not shown). The results of these assays are consistent with the induction of a CC49 anti-idiotype antibody response targeting the TAG-72 binding domain of the CC49-ζ CAR, with onset in a majority of treated patients following the second or third infusion of 10^10^ CART72 cells.

### CART72 persistence

**Fig. 4:**
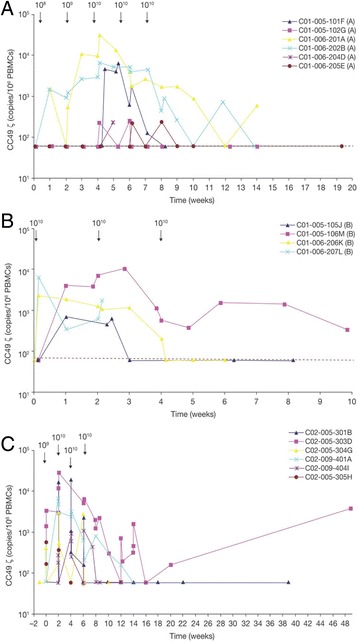
CART72 persistence in blood. The CC49-ζ copy number measured by quantitative PCR and expressed per 10^6^ circulating PBMCs over time is shown in: (**a**) Part A dose escalation phase of CART72 cells administered by IV infusion in trial C-9701; (**b**) Part B expansion phase of trial C-9701; and (**c**) CART72 cells administered by HA infusion in trial C-9702. HA, hepatic artery; PBMC, peripheral blood mononuclear cell; PCR, polymerase chain reaction

CART72 cells were detected by DNA quantitative polymerase chain reaction (PCR) analysis in the blood of all patients post-infusion (Fig. 4). In trial C-9701 (Part A) peak levels occurred at weeks 4 to 6 following the first infusion of 10^10^ CART72 cells and ranged from 220 to 31,418 copies/10^6^ peripheral blood mononuclear cells (PBMCs) (Fig. 4a). In trial C-9701 (Part B) peak levels occurred at weeks 0 or 2 following the first or second infusion of 10^10^ CART72 cells and ranged from 682 to 10,014 copies/10^6^ PBMCs (Fig. 4b). Six of nine patients showed peak levels representing greater than 0.1% of PBMCs. Rapid clearance of CART72 cells from the blood was observed following the second or third infusion of 10^10^ CART72 cells in five of 10 patients. In three patients, there was a 1- to 2-log decrease in the level of CART72 cells following the second 10^10^ cell infusion, and CART cells were detected intermittently at low levels thereafter for at least 10 weeks following the first infusion. Two patients were not evaluable for clearance assessment because of short follow-up. In general, clearance of CART72 cells coincided with the development of the CC49-ζ idiotype antibody response.

In trial C-9702, all patients had detectable levels of CART72 by DNA PCR in the blood following HA infusion of 10^10^ CART72 cells (Fig. 4c). In contrast to trial C-9701, peak levels occurred variably after the second, third or fourth infusion of 10^10^ CART72 cells and ranged from 1157 to 27,917 copies/10^6^ PBMCs. Four of six patients demonstrated clearance of CART72 cells by Week 6 to 8 and a fifth by Week 14. The final patient (303D) showed persistence of CART72 cells through 48 weeks of follow-up despite early detection of an anti-idiotype response, with rising levels from Week 16 to 48, possibly associated with loss in titer of the inhibitor. Of note, this patient showed the greatest initial CART72 expansion (1369 to 27,917 copies/10^6^ PBMCs from Day 1 to 15) associated with an acute febrile syndrome consistent with cytokine release from Week 2 to 3, as well as a recurrent syndrome consistent with cytokine release two months following the final infusion at Week 16, associated with an increase in the level of CART72 in his blood. In the face of evolving evidence of rapid CART72 clearance in most patients associated with generation of an anti-CC49 idiotype response, a decision was made to eliminate co-administration of IFN-α from the last two patients (305H and 404I) based on the hypothesis that IFN-α might have exacerbated immunogenicity of the CART72 cells. Development of an anti-CC49 antibody response associated with rapid CART72 clearance was still observed at Weeks 4 and 8 in these last two patients without IFN-α co-treatment.

### Tumor trafficking of CART72 Cells

Three patients across both trials underwent paired screening and on-treatment tumor biopsies to measure TAG-72 expression levels and tumor trafficking of CART72 cells (Table 3). In one patient in trial C-9701, baseline expression of TAG-72 was evident in 5% of cells in a rectal metastasis, as measured by immunohistochemistry. A rectal tumor mass biopsied 3 days following the third infusion of 10^10^ CART72 cells showed 10% TAG-72 expression. Although CART72 cells were still detectable in blood (1704 copies/10^6^ PBMCs) at the time of biopsy, CART72 cells were undetectable in the rectal tumor. Two patients in trial C-9702 underwent on-treatment biopsies of liver metastases 1 day and 2 weeks following the fourth CART72 infusion. In the former, CART72 cells were detectable in both the blood and tumor tissue (1937 and 220 copies/10^6^ PBMCs, respectively) and the biopsied tumor showed an infiltrate of CD3+ T cells. In contrast, no CART72 cells were detected in blood or tumor of the second patient biopsied at the later time point. Tumor TAG-72 expression levels are not available on these latter two patients.

**Table 3:** CART72 levels in tumor biopsies

Patient ID	CART72 delivery	Tumor biopsy location	Timing of biopsy	CC49-ζ by PCR (copies/10^6^ cells)	
				Tumor	Blood
303D	HA	Liver	Day of 4th infusion	220	1937
301B	HA	Liver	2 weeks post 4th infusion	<50	<117
201A	IV	Rectum	Day of 5th infusion	<50	1704

*HA* hepatic artery, *IV* intravenous, *PCR* polymerase chain reaction

Levels of CC49-ζ modified CART72 cells were measured by PCR in tumor biopsies taken at variable time points post-CART72 infusion in three patients. CC49-ζ levels in biopsied tumor tissue were compared to levels in blood at corresponding time points. Patients 303D and 301B were treated in trial C-9702 and patient 201A was treated in trial C-9701

**Fig. 5:**
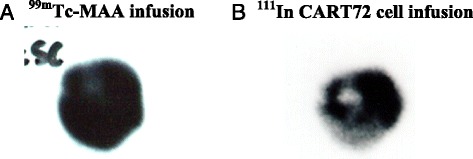
Tumor trafficking of ^111^In-labeled CART72 cells. ^111^In-labeled CART72 cells were infused in four patients and compared to infusion of ^99m^Tc-MAA and imaging was performed at 3–96 hours post-infusion. Representative images from one patient at 24 hours post infusion of 10^10^ CART72 cells are notable for decreased penetration of ^111^In-labeled CART72 cells (**b**) into the center of a liver metastases compared with well distributed dissemination of ^99m^Tc-MAA (**a**). ^111^In, ^111^Indium; ^99m^Tc-MAA, technetium albumin aggregated particles

Four patients with radiologic liver metastases in trial C-9701 were enrolled in a substudy to monitor the in vivo trafficking of ^111^Indium-labeled CART72 cells based on experience with this technique to monitor trafficking of adoptively transferred tumor-infiltrating lymphocytes [24, 25]. Trafficking of ^111^Indium-labeled CART72 cells was compared to tumor uptake of infused technetium albumin aggregated particles (^99m^Tc-MAA), which visualize vascularized tumor tissue. In general, CART72 cells were observed to cluster at the perimeter of large tumor masses without evidence of deep tumor penetration, whereas ^99m^Tc-MAA distributed uniformly throughout the metastatic tumors consistent with adequate vascularization of the tumor masses (Fig. 5).

### Clinical outcome

No radiologic tumor responses were observed in either trial during the planned 12-week monitoring period. The best response in both trials was progressive disease. One of 10 patients (207 L) in trial C-9701 demonstrated a > 50% decrease in CEA and 2 additional patients (201A, 205E) demonstrated transient reductions in CEA of unclear significance (Fig. 3d, e). One of 6 patients (303D) in C-9702 demonstrated two transient decreases in CEA levels concordant with clinical febrile syndromes suggestive of CRS (Fig. 3f). Patients were not routinely monitored for possible late tumor regressions following early radiologic progression as has been reported in more recent trials of cancer immunotherapies [25]. One patient (303D) treated with intra-HA infusion of CART72 cells remained alive with persistently detectable and rising levels of CART72 cells in blood 48 weeks following initial treatment. This patient demonstrated the greatest acute CART72 expansion, had symptoms consistent with CRS on two separate occasions, both of which were associated with transient drops in serum CEA levels, and had the longest duration of CART72 persistence (48+ weeks).

## Discussion

This report describes the results of a pair of important early clinical trials in the field of CAR-T cells targeting solid tumors. These trials preceded the next clinical trial of CAR-T cells in a solid tumor indication by more than 5 years [29]. Initially dismissed as negative studies, the lessons learned retain value and are especially relevant now given the resurgence of interest in this field. Important observations from these trials include: 1) the robustness and reproducibility of autologous CD4 and CD8 T cell transduction and expansion using a gamma-retroviral vector and co-stimulation with CD3xCD28 immunomagnetic beads; 2) persistence of CART72 cells in blood (up to 48 weeks in one subject) with evidence of clinical symptoms consistent with mild to moderate CRS, most pronounced in patients with the highest levels of CART72 cells in blood; 3) trafficking of CART72 cells, at least to some degree, to tumor tissues; and 4) most importantly, the immunogenicity of the CART72 cells, despite the use of a human chimeric CC49 scFv in the CAR construct, that was associated with rapid clearance of subsequent CART72 infusions in most patients.

The CART72 manufacturing process used in this study was nearly identical to that previously described for the production of CD4-ζ CAR-T cells from HIV-infected patients [11, 12] using CD3xCD28 bead co-stimulation in the presence of low-dose IL-2 (200 IU/mL). The efficiency and consistency of ex vivo T cell transduction and expansion from CRC patients, reaching 4–5 x 10^10^ total T cells within 17 days, was remarkably similar to that reported for manufacturing of CD4-ζ CAR-T cells from HIV-infected patients and from healthy donors [9]. High transduction efficiencies of approximately 40% using the *kat*293 high-titer gamma-retroviral vector were reported for both autologous CAR-T products and yielded mixed populations of gene-modified CD4 and CD8 T cells. This high level of transduction efficiency eliminated the need for post-transduction purification to enrich for transduced cells, thus significantly simplifying the manufacturing process. Phenotypic analysis revealed a high proportion of activated T cells expressing CD62L and CD28, with intermediate expression of CD25, and resulted in engineered T cells resistant to activation induced cell death upon TAG-72 engagement.

In distinct contrast to the clinical experience with CD4-ζ CAR-T cells, which persisted at relatively stable levels in the blood of treated HIV-infected patients for up to 10 years [11–13], rapid clearance of CART72 cells from the blood following repeated infusions of 10^10^ cells was observed in most patients treated in these cancer studies. This clearance was associated with the development of an anti-idiotype immune response directed against the huCC49 scFv, initially identified via interference with measurement of sera TAG-72 levels in a CC49 capture RIA. Fortuitously, in retrospect, the TAG-72 RIA assay served as a robust assay to measure the onset and kinetics of induction of this CC49-ζ anti-idiotype antibody response. These results provide a cautionary note to others in the field of CAR-T cell therapies seeking to target tumors other than malignant B cells or plasma cells with scFv-based CAR constructs. However, CC49 may have particularly immunogenic antigen-binding epitopes. Following initiation of these CART72 trials, it was reported that anti-idiotype responses were part of the HAMA response in 54% of the patients treated with muCC49 [30]. Subsequent efforts led to the creation of next-generation huCC49 antibodies with only the specificity-determining residues of the mouse TAG-72 binding epitopes grafted onto the frameworks of the human antibody [31]. One such variant, V59, was shown not to react with the anti-idiotype present in the sera of patients treated with muCC49. This next-generation huCC49 construct might be a better choice for future CAR-T cell programs targeting TAG-72. Alternatively, CAR-T cell constructs using fully human extracellular antibody domains should be considered. Finally, patients in these CART72 trials did not receive lymphodepleting chemotherapy prior to infusion of CART72 cells, as the beneficial effect of these conditioning regimens on the in vivo proliferation and clinical activity of adoptively transferred tumor-specific T cells had not yet been reported [32]. It is possible that lymphodepleting chemotherapy (eg, fludarabine and cyclophosphamide), now routinely administered with second-generation CAR-T cell therapies, will further reduce the risk of developing anti-CAR immune responses.

Despite the induction of an anti-CAR immune response and more rapid clearance of CART72 cells in these trials than observed with CD4-ζ CAR-T cells, some encouraging preliminary signals of in vivo tumor trafficking and biologic activity were observed. Short-term detection of CART72 cells in blood was observed in most patients. Several patients treated with direct HA infusion of CART72 cells developed a constellation of clinical symptoms, which, in retrospect, was consistent with CRS. One patient with the highest peak levels of CART72 cells in blood developed symptoms consistent with CRS associated with transient drops in serum CEA levels, and showed rising levels of CART72 cells at 48 weeks post-first infusion. In one of three patients, CART72 cells were detected in a biopsy of a liver metastasis post-infusion, confirming trafficking of cells to tumor tissue. However, ^111^Indium-labeling of infused CART72 cells suggested incomplete penetration of CART72 cells into the center of large tumor masses in several patients analyzed. Future CAR-T cell programs in advanced solid tumors may benefit from incorporating strategies to enhance homing and tumor penetration of infused CAR-T cells, or focus on patients with minimal tumor burden following cytoreductive therapies. In addition, second-generation CAR T-cell approaches incorporating additional T-cell signaling domains (eg, CD28, 4-1BB) have been shown to enhance in vivo CAR-T cell expansion and persistence, and have resulted in dramatic clinical responses in B-cell malignancies [33–37]. Preclinical data also support the use of ICOS-ζ intracellular domains to promote bipolar helper T (Th)1/Th17 cell differentiation, resulting in improved effector function and in vivo persistence [38]. One or more of these additional T-cell co-stimulatory domains will likely be incorporated into all future solid tumor CAR-T cell programs.

An additional challenge to the development of next-generation CAR-T cells targeting solid tumors remains the limited number of truly tumor-restricted cell-surface targets, posing a clinical risk for on-target/off-tumor toxicity [39]. No serious on-target/off-tumor toxicity was observed in these CART72 trials suggesting that TAG-72 remains a reasonable CAR target for next-generation solid tumor approaches. However, greater in vivo proliferation, effector function or persistence of second- and third-generation CAR-T cells may uncover toxicities not observed in these first-generation trials. Incorporation of titratable control systems to regulate and/or eradicate CAR-T cells if unacceptable toxicity arises should be considered [40, 41]. Heterogeneous tumor-associated surface antigen expression in most solid tumors also remains a vexing problem. Tumor-associated TAG-72 expression is non-uniform and there were insufficient data from these CART72 trials to know whether co-administration of IFN-α resulted in sufficient TAG-72 upregulation to avoid antigen-loss escape. Targeting of multiple tumor antigens concurrently with multi-specific CAR-T cells is of interest for future solid tumor CAR-T programs [5, 42, 43].

## Conclusions

The field of CAR-T cells has established clear clinical proof-of-concept for treatment of B-cell malignancies. These belated clinical results from the first CAR-T cell trials conducted in a solid tumor indication provide valuable insights to guide the design of next-generation CAR-T cell approaches tackling the more complex tumor microenvironment of solid tumors.

## Abbreviations

99mTc-MAA: Technicium Tc 99 m albumin aggragated injection
B72.3: murine antibody recognizing a CC49 non-competing epitope of TAG-72)
CAR: chimeric antigen receptor
CART72 cells: T cells transduced with the CC49-zeta CAR
CD3xCD38 beads: anti-CD3 and anti-CD28 antibody conjugated beads
CEA: carcinoembryonic antigen
CRC: colorectal cancer
CTCAE 2.0: Common Terminology Criteria for Adverse Events Version 2.0
HA: hepatic artery
HAMA: human anti-mouse antibody
HIV: human immunodeficiency virus
HRP: horse radish peroxidase
huCC49: human chimeric antibody directed against TAG-72
ICOS: inducible T cell costimulatory (CD278)
IFN: interferon
*kat*293: high efficiency gamma-retroviral transduction system
muCC49: murine antibody directed against TAG-72
NHS: normal human serum
PBMC: peripheral blood mononuclear cells
PCR: polymerase chain reaction
RCR: replication competent retrovirus
RIA: radioimmunoassay
scFv: single chain variable fragment
TAG-72: Tumor associated glycoprotein-72
TCR: T cell receptor
ULN: upper limit of normal
